# Precision Prevention for Health: The Global Alliance Framework for the Promotion of Physical Activity

**DOI:** 10.1007/s40279-026-02495-4

**Published:** 2026-09-21

**Authors:** Jürgen M. Steinacker, Willem van Mechelen, Lars Engebretsen, Evert Verhagen, Mats Börjesson, Marcela González-Gross, Mark R. Hutchinson, Petra Zupet, Herbert Löllgen, Dina C. Janse van Rensburg, Norbert Bachl, Sergio Migliorini, Pieter D‘Hooghe, Christian Schneider, Jiao Jiao, Ka Ki Wong, Jeroen Swart, Leigh Gordon, Victoria Badtieva, Borja Muniz-Pardos, Tony Lei Shi, Li Xiangchen, Feng Qiang, Guoping Li, Minhao Xie, Xia Zhang, Uğur Erdener, Federica Fagnani, Fabio Pigozzi, Chiara Fossati, Luigi Di Luigi, Francisco Arroyo, Jean-François Kaux, Camille Tooth, Shulin Cheng, Xiuqiang Wang, Emin Ergen, Bülent Ülkar, Frederic Dutheil, Ruediger Reer, Theodora Papadopoulou, Clea Papaellina, Demitri Constantinou, Jason Constantinou, Merling Phaswana, Mamosilo Lichaba, Philippe Gradidge, Mandisa Simamane, Georgia Torres, Natalia Neophytou, Elelwani D. Nevhufumba, Bernhard Schwaab, José Antonio Casajús Mallen, Martin Halle, Martine Duclos, Daniel Neunhaeuserer, Andrea Ermolao, Francesca Battista, Federica Duregon, Julio Motta-Pensabene, Yiannis Koutedakis, Lena Tepohl, Wilhelm Bloch, Nicolaas P. Pronk, Anastasia Fischer, Lisset Pantoja-Arévalo, Ana V. Cintrón, Adewale S. Adebero, Jill Borresen, Johannes Zwerver, Diana M. Ramos-Caballero, Erica M. Mancera-Soto, Pedro Manonelles, Benedict Tan, Ingrid Burrowes, Carl Ward, Kim R. Quimby, Christina Howitt, Robert Clive Landis, Hans Hauner, Mikio Hiura, Luz Divina De La Cruz Lastre, Gunnar Treff, Fotini Tsofliou, José Kawazoe Lazzoli, Umutcan Kayıkçı, Mourad Ghrairi, Vincent Onywera, Robert Ojiambo, Robert Sallis, Sophia Papamichalopoulos, Maher Zahar, Christine Joisten, Bernd Wolfarth, Sandra Rozenstoka, Hashel al Tunaiji, Kiera Wason, Felix Drummond, Wolfgang Schobersberger, Vahur Ööpik, Eve Unt, Anna Jegier, Stéphane Doutreleau, Seung Hoon Han, Jörg F. Debatin, Nils Schumacher, Luís B. Sardinha, Lenka Dienstbach-Wech, Tuba Melekoğlu, Sheng Zeng, Maurizio Casasco, Kristy Howells, Eva-Maria Klinkisch, James Bilzon, Gregory Heath, Thomas Horstmann, Eduardo H. De Rose, Nick Webborn, Klaus-Michael Braumann, Li Jing, Xiao Xu, Elizabeth Moxley, Yi Zheng, Alison V. September, Malcolm Collins, Olena Andrieieva, Svitlana Drozdovska, Andreas Michael Nieß, Rikke Helene Moe, Carolin Knoke, Silvano Zanuso, Francesco Zanatta, Veronika Všetíčková, Spyridon Zormpas, Christoforos D. Giannaki, Brian P. Carson, Matthew P. Herring, Ciara McCormack, Marco Vecchiato, Thomas Yvert, Makro Korenjak, Benjamin Billet, Andréia Lópes Azevedo, Naama Constantini, Sigmund Loland, Aaron Leigh Baggish, Luis Monteiro, Nuray Yazihan, Yannis P. Pitsiladis

**Affiliations:** 1 European Initiative for Exercise in Medicine (EIEIM), Ulm, Germany; 2 Institute for Rehabilitation Medicine Research at Ulm University, Bad Buchau, Germany; 3 International Federation of Sports Medicine, Fédération Internationale de Médecine du Sport (FIMS), Lausanne, Switzerland; 4 Department of Public and Occupational Health, Amsterdam UMC, Location VUmc, Amsterdam, The Netherlands; 5 School of Human Movement and Nutrition Sciences, Faculty of Health and Behavioural Sciences, University of Queensland, Brisbane, Australia; 6 Health Through Physical Activity, Lifestyle and Sport Research Centre, Department of Human Biology, Faculty of Health Sciences, University of Cape Town, Cape Town, South Africa; 7 UCD School of Public Health, Physiotherapy and Population Sciences, University College Dublin, Dublin, Ireland; 8 Division of Orthopedic Surgery, University of Oslo, Oslo, Norway; 9 Institute of Medicine, Department of Molecular and Clinical Medicine, Sahlgrenska Academy, University of Gothenburg, Gothenborg, Sweden; 10 Center for Lifestyle Intervention, Department of Medicine, Geriatric and Emergency Care, Sahlgrenska University Hospital, Region Västra Götaland, Gothenburg, Sweden; 11 ImFINE Research Group, Department of Health and Human Performance, Facultad de Ciencias de La Actividad Física y del Deporte, Universidad Politécnica de Madrid, Madrid, Spain; 12 Exercise Is Medicine Spain, University of Zaragoza, Saragossa, Spain; 13 Orthopaedics and Sports Medicine, University of Illinois at Chicago, Chicago, USA; 14 ACSM Foundation, ACSM, Indianapolis, USA; 15 European Federation of Sports Medicine Associations, Lausanne, Switzerland; 16 IMS Institute for Medicine and Sports, Ljubljana, Slovenia; 17 Section Sports Medicine and SEMLI, Faculty of Health Sciences, University of Pretoria, Pretoria, South Africa; 18 Medical Advisory Panel, World Netball, Manchester, UK; 19 Institute of Sports Science, University of Vienna, Vienna, Austria; 20 Austrian Institute of Sports Medicine, Vienna, Austria; 21 World Triathlon Medical Committee, World Triathlon, Lausanne, Switzerland; 22 Aspetar, Orthopaedic and Sports Medicine Hospital, Doha, Qatar; 23 Orthopaedic Center Theresie, Munich, Germany; 24 Department of Sports and Health Sciences, Hong Kong Baptist University, Hong Kong, SAR China; 25 UCT Health through Physical Activity, Lifestyle and Sport Research Centre (HPALS), Cape Town, South Africa; 26 I M Sechenov First Moscow State Medical University, Moscow, Russia; 27 Moscow Centre for Research and Practice in Medical Rehabilitation, Restorative and Sports Medicine of Moscow Healthcare Department, Moscow, Russia; 28 EXER-GENUD (Growth, Exercise, NUtrition and Development) Research Group (S72_23R), FIMS Collaborating Center of Sports Medicine, University of Zaragoza, Saragossa, Spain; 29 School of General Education, Beijing Normal-Hong Kong Baptist University, Zhuhai, China; 30 China Institute of Sport Science, General Administration of Sport of China, Beijing, China; 31 China National Institute of Sports Medicine (NISM), Beijing, China; 32 Asian Federation of Sports Medicine, Hong Kong, China; 33 SportAccord, Lausanne, Switzerland; 34 Department of Movement, Human and Health Sciences, University of Rome ‘Foro Italico’, Rome, Italy; 35 FIMS Collaborating Center of Sports Medicine, Guadalajara, Mexico; 36 ReFORM IOC Research Centre for Prevention of Injury and Protection of Athlete Health, Liège, Belgium; 37 Service de Médecine Physique, Réadaptation et Traumatologie du Sport (SportS2), FIFA Medical Centre of Excellence, FIMS Collaborative Centre of Sports Medicine, CHU de Liège, Liège, Belgium; 38 Luxembourg Institute of Research in Orthopedics, Sports Medicine and Science (LIROMS), Luxembourg, Luxembourg; 39 Exercise Translational Medicine Centre, Global Institute of Future Technology and Institute of Translational Medicine, Shanghai Jiao Tong University, Shanghai, China; 40 Sports Medicine and Performance Organization, Gloria Sports Arena, Belek, Antalya, Türkiye; 41 Sports Medicine Department, Faculty of Medicine, Ankara University, Ankara, Turkey; 42 Université Clermont Auvergne, CNRS, LaPSCo, CHU Clermont-Ferrand, Clermont-Ferrand, France; 43 Department of Movement Science, University of Hamburg, Hamburg, Germany; 44 Defence Medical Rehabilitation Centre (DMRC), Loughborough, UK; 45 Department of Exercise Science and Sports Medicine, Faculty of Health Sciences, University of the Witwatersrand, Johannesburg, South Africa; 46 Hacettepe University, Ankara, Türkiye; 47 German Society for the Prevention and Rehabilitation of Cardiovascular Diseases (Deutsche Gesellschaft für Prävention und Rehabilitation Von Herz-Kreislauferkrankungen (DGPR)), Koblenz, Germany; 48 Curschmann Clinic, Rehabilitation Hospital for Cardiology, Angiology and Diabetes, Timmendorfer Strand, Germany; 49 GENUD “Growth, Exercise, NUtrition and Development” Research Group, University of Zaragoza, Saragossa, Spain; 50 Department of Physiatry and Nursing, Faculty of Health and Sport Science (FCSD), University of Zaragoza, Saragossa, Spain; 51 European Association of Preventive Cardiology (EAPC), European Society of Cardiology (ESC), Biot, France; 52 Department of Preventive Sports Medicine and Sports Cardiology, TUM School of Medicine and Health, Technische Universität München, Munich, Germany; 53 INRAE, UNH, CHU Clermont-Ferrand, Department of Sport Medicine and Functional Exploration, Université Clermont Auvergne, Clermont-Ferrand, France; 54 Sports and Exercise Medicine Division, Department of Medicine, Università degli Studi di Padova, Padua, Italy; 55 Exercise is Medicine Italy, Università degli Studi di Padova, Padua, Italy; 56 SportsMed Clínic and High Performance, Guatemala City, Guatemala; 57 Thessaly University, Volos, Greece; 58 Institute for Cardiovascular Research and Sport Medicine, German Sport University, Cologne, Germany; 59 HealthPartners Institute, Minneapolis, MN USA; 60 Division of Sports Medicine, Nationwide Children’s Hospital, Columbus, OH USA; 61 The Ohio State University College of Medicine, Columbus, OH USA; 62 Department of Physical Medicine, Rehabilitation and Sports Medicine, University of Puerto Rico School of Medicine, San Juan, PR USA; 63 Puerto Rico Sports Medicine Federation, San Juan, PR USA; 64 Pan American Confederation of Sports Medicine, San Juan, PR USA; 65 School of Public and Allied Health, Prairie View A&M University, Prairie View, TX USA; 66 Sport, Exercise Medicine and Lifestyle Institute, Faculty of Health Sciences, University of Pretoria, Pretoria, South Africa; 67 University Medical Center Groningen, Center for Human Movement Sciences, University of Groningen, Groningen, The Netherlands; 68 Sports Valley, Gelderse Vallei Hospital, Ede, The Netherlands; 69 School of Medicine and Health Sciences, Universidad del Rosario, Bogotá, Colombia; 70 Departamento del Movimiento Corporal Humano, Facultad de Medicina, Universidad Nacional de Colombia, Bogotá, Colombia; 71 Department of Sports Medicine, San Antonio Catholic University of Murcia, Spanish Society of Sports Medicine, Murcia, Spain; 72 Exercise is Medicine, Singapore National Centre, Singapore, Singapore; 73 Barbados Sports Medicine Association (BSMA), Bridgetown, Barbados; 74 The George Alleyne Chronic Disease Research Centre, Caribbean Institute for Health Research, The University of the West Indies, Cave Hill Campus, Cave Hill, Barbados; 75 Edmund Cohen Laboratory for Vascular Research, The George Alleyne Chronic Disease Research Centre, Caribbean Institute for Health Research, The University of the West Indies, Cave Hill Campus, Cave Hill, Barbados; 76 Else Kröner Fresenius Centre for Nutritional Medicine, School of Medicine and Health, Technical University of Munich and German Diabetes Foundation, Düsseldorf, Germany; 77 Research Center for Brain and Health Sciences, Aomori University, Aomori, Japan; 78 Exercise is Medicine Japan, Tokyo, Japan; 79 Universidad Nacional de Colombia, Bogotá, D.C. Colombia; 80 Institute of Molecular Sports Medicine and Rehabilitation, Paracelsus Medical Private University, Salzburg, Austria; 81 Ludwig Boltzmann Institute for Digital Health and Prevention, Salzburg, Austria; 82 School of Life and Health Sciences, University of Roehampton, London, SW15 4JD UK; 83 Biomedical Institute, Medical School at Fluminense Federal University, Niterói, Brazil; 84 Cardiology Division, Santa Teresa Hospital, Petrópolis, Brazil; 85 Division of Maternal Fetal Medicine, Department of Obstetrics and Gynaecology, Faculty of Medicine, Hacettepe University, Ankara, Turkey; 86 HMS FIFA Medical Centre of Excellence, Dubai, UAE; 87 Division of Research, Innovation and Outreach, KCA University, Nairobi, Kenya; 88 Division of Medical Education, Department of Physiology and Biophysics, Weill Cornell Medicine Qatar, Ar-Rayyan, Qatar; 89 Kaiser Permanente Bernard J Tyson School of Medicine, Pasadena, CA USA; 90 Department of Visceral Surgery, Spital Männedorf Hospital, Männedorf, Switzerland; 91 Oral Health Unit, Tunisian Centre of Sports Medicine and Science, Tunis, Tunisia; 92 German Association for Sports Medicine and Prevention (Deutsche Gesellschaft für Sportmedizin und Prävention (DGSP)), Frankfurt, Germany; 93 Institute of Movement and Neuroscience, German Sport University Cologne, Cologne, Germany; 94 Department of Sports Medicine, Humboldt University and Charité University School of Medicine, Berlin, Germany; 95 Sports Laboratory, Sports Medicine and Physical Health Centre, Riga, Latvia; 96 Rīga Stradiņš University, Riga, Latvia; 97 Sport Medicine and Sciences Unit, Zayed Military University, Abu Dhabi, United Arab Emirates; 98 UAE NOC Sport Medicine Committee, UAE NOC, Dubai, United Arab Emirates; 99 Department of Physical Medicine and Rehabilitation, Zayed Military Hospital, Abu Dhabi, United Arab Emirates; 100 EuropeActive, Rue du Commerce 20, Bruxelles 1000, 1040 Brussels, Belgium; 101 Instituto de Medicina do Esporte (IME), Porto Alegre, Brazil; 102 Institute for Sport Medicine, Alpine Medicine and Health Tourism (ISAG), UMIT TIROL Private University for Health Sciences and Technology GmbH, Hall and University Hospital, Innsbruck, Austria; 103 Institute of Sport Sciences and Physiotherapy, Faculty of Medicine, University of Tartu, Tartu, Estonia; 104 Estonian Sports Medicine Federation, Tartu, Estonia; 105 Department of Sports Medicine and Rehabilitation, Institute of Clinical Medicine, University of Tartu, Tartu, Estonia; 106 Department of Sports Medicine, Medical University of Lodz, Lodz, Poland; 107 Unité Sport Santé Prévention, CHU de Grenoble Alpes, La Tronche, France; 108 Laboratoire TIMC-CNRS, Université Grenoble Alpes, La Tronche, France; 109 Department of Rehabilitation Medicine, Hanyang University College of Medicine, Seoul, Republic of Korea; 110 Science and Transfer Center Active City, University of Hamburg, Hamburg, Germany; 111 Exercise and Health Laboratory, CIPER, Faculdade de Motricidade Humana, Universidade de Lisboa, Lisbon, Portugal; 112 Department of Orthopaedics, Spine and Trauma Surgery, Hospital zum Heiligen Geist Frankfurt, Frankfurt, Germany; 113 Faculty of Sport Sciences, Akdeniz University, Antalya, Turkey; 114 Key Laboratory of Skeletal Regeneration of Hainan Province, Haikou, China; 115 Italian Federation of Sports Medicine (FMSI), Rome, Italy; 116 International Association of Physical Education in Higher Education (AIESEP), Liège, Belgium; 117 Canterbury Christ Church University, Canterbury, UK; 118 Catholic University of Applied Sciences, Karlstraße, Freiburg im Breisgau, Germany; 119 Centre for Clinical Rehabilitation and Exercise Medicine (CREM), Centre for Sport Exercise and Osteoarthritis Research (CSEO), Department for Health, University of Bath, Claverton Down, Bath, UK; 120 UTHSC College of Medicine Chattanooga, University of Tennessee at Chattanooga, Chattanooga, TN USA; 121 Department of Health and Sport Sciences, TUM School of Medicine and Health, Technische Universität München, Georg-Brauchle-Ring, Munich, Germany; 122 School of Sport, Exercise and Health Sciences, Loughborough University, Loughborough, UK; 123 Department of Sport and Exercise Medicine, Faculty of Psychology and Human Movement Science, University of Hamburg, Hamburg, Germany; 124 National Team Medicine Administration, China Institute of Sports Medicine, Beijing, China; 125 MGI Tech, Shenzhen, China; 126 School of Nursing, Northern Illinois University, DeKalb, IL USA; 127 Faculty of Psychology and Educational Sciences, University of Geneva, Geneva, Switzerland; 128 Institute for Sustainable Development Goals, Tsinghua University, Beijing, China; 129 Health through Physical Activity Lifestyle and Sport Research Centre (HPALS) and Division of Physiological Sciences, Department of Human Biology, University of Cape Town, Cape Town, South Africa; 130 International Federation of Sports Medicine (FIMS) Collaborative Centre of Sports Medicine, University of Cape Town, Cape Town, South Africa; 131 National University of Ukraine on Physical Education and Sport, Kyiv, Ukraine; 132 Department of Sport Medicine, Medical Clinic, University Hospital of Tuebingen, Tuebingen, Germany; 133 Interfacultary Research Institute of Sports and Physical Activity, University of Tuebingen, Tuebingen, Germany; 134 European Alliance of Associations for Rheumatology (EULAR), Zurich, Switzerland; 135 Center for Treatment of Rheumatic and Musculoskeletal Diseases (REMEDY), Diakonhjemmet Hospital, Oslo, Norway; 136 Department of Psychiatry and Psychotherapy II, Ulm University, Ulm, Germany; 137 Exercise Is Medicine Germany, Frankfurt, Germany; 138 Karlsruhe Institute of Technology, Karlsruhe, Germany; 139 European Liver Patients’ Association (ELPA), Brussels, Belgium; 140 Greek Carers Network EPIONI, Athens, Greece; 141 School of Life and Health Sciences, University of Nicosia, Nicosia, Cyprus; 142 Exercise is Medicine Ireland National Centre, University of Limerick, Limerick, Ireland; 143 Department of Sport Science and Nutrition, Faculty of Science and Engineering, Maynooth University, Kildare, Ireland; 144 European Network of Outdoor Sports (ENOS), Annemasse, France; 145 French Ministry of Sports and National Outdoor Centre, Annemasse, France; 146 POLIS Network, Brussels, Belgium; 147 Shaare Zedek Medical Center, Hebrew University, Jerusalem, Israel; 148 Department of Sport and Social Sciences, Norwegian School of Sport Sciences, Oslo, Norway; 149 Swiss Olympic Medical Center, Lausanne University Hospital (CHUV), Lausanne, Switzerland; 150 Institute for Sport Science, University of Lausanne (ISSUL), Lausanne, Switzerland; 151 Pathophysiology Department, Faculty of Medicine, Ankara University, Ankara, Turkey; 152 Interdisciplinary Food, Metabolism and Clinical Nutrition Department, Institute of Health Sciences, Ankara University, Ankara, Turkey; 153 Berlin Institute of Public Health, Charité-Universitätsmedizin Berlin, Berlin, Germany; 154 Technogym, Medical and Scientific Department, Technogym and Wellness Foundation, Milan, Italy; 155 Edith Cowan University, Perth, Australia; 156 Wellness Foundation, Milan, Italy; 157 Department of Psychology, University of Milano-Bicocca, Milan, Italy; 158 Centre for Exercise Science and Medicine (CESAME), Faculty of Science, Hong Kong Baptist University, Hong Kong, SAR China; 159 University of Mainz, Mainz, Germany; 160 Department of Sport Science and Physical Education, Faculty of Health and Sport Science, University of Agder, Kristiansand, Norway

## Abstract

This conceptual, implementation-oriented paper proposes a framework for precision prevention centred on physical activity promotion and early risk mitigation, aiming to support a shift from reactive disease management toward proactive prevention. The proposed framework focuses on the early identification of risk trajectories by integrating biological, environmental, and social determinants of health through systems thinking, multi-omics technologies, digital health, and behavioural science. It aims to inform tailored, context-sensitive interventions that will help to mitigate chronic non-communicable diseases (NCDs), while supporting lifelong resilience. Within this framework, physical activity is positioned as the central, modifiable anchor behaviour through which personalised prevention strategies may be operationalised. Building on the 2025 Hamburg Declaration and the Global Alliance for the Promotion of Physical Activity’s (GA@PA) mission, this conceptual paper proposes a multidimensional model, combining personalised strategies for physical activity, nutrition, sleep, and mental health with contextual environmental adaptations. By bringing together stakeholders across multiple sectors, including healthcare, education, urban planning, sport organisations, industry, and community groups, GA@PA aims to strengthen global collaboration and to accelerate the implementation of evidence-based strategies for physical activity promotion. Emerging technologies, including digital twins, wearable biosensors and artificial intelligence (AI)-driven analytics, are discussed as potential enabling tools for real-time monitoring and for providing input for adaptive interventions, while mobile health platforms may extend access to underserved populations. Rather than providing prescriptive solutions, the paper outlines key implementation fundamentals. These include technological infrastructure with interoperable biosensors and AI aligned with prevailing ethical frameworks such as the AI Act and UNESCO recommendations; interdisciplinary research on resilience and outlier populations; policy and education reform, including routine assessment of physical activity as a vital sign within healthcare encounters; and cross-sector collaboration spanning urban design to sport-for-development initiatives such as the International Olympic Committee (IOC)’s Olympism365. Sports organisations may support broader participation through sport-for-all programmes. The framework emphasises equity, cultural adaptation, and community co-design as critical features for scalability across diverse global contexts, highlighting the role of sport, fitness, and public health partnerships in creating a context where healthy choices become accessible and sustainable. Overall, this conceptual paper outlines a practice-informed framework proposing precision prevention as an evolving and testable implementation framework for supporting sustainable and equitable prevention systems. By integrating science, technology and policy through a multisectoral lens, precision prevention is proposed as a testable pathway to reduce the NCD burden, to enhance the health span and to foster resilient societies.

**Supplementary Information:**

The online version contains supplementary material available at https://doi.org/10.1007/s40279-026-02495-4.

## Key Points

**Table:** 

Precision prevention proposes a personalised, data-informed framework to promote physical activity and to prevent non-communicable diseases across the life course.
The Global Alliance for the Promotion of Physical Activity (GA@PA) framework integrates biological, behavioural, contextual, and digital determinants to support scalable and context-sensitive prevention strategies
Physical activity is positioned as a core, modifiable behaviour, supported by emerging technologies, education, and policy alignment.
Physical activity, exercise, and sport may support healthier lives when implemented within supportive social and environmental contexts
Effective implementation of physical activity and exercise interventions requires cross-sector collaboration, workforce development, and attention to equity and real-world feasibility.
Physical activity is proposed as the central behavioural target through which precision prevention may be operationalised.

## Introduction

This publication builds upon the Hamburg Declaration [1]; i.e. the inaugural consensus statement of the Global Alliance for the Promotion of Physical Activity (GA@PA), which identified physical inactivity as one of the most significant and modifiable public health challenges of the twenty-first century, contributing substantially to global morbidity and mortality [2]. Grounded in substantial epidemiological evidence linking physical (in)activity behaviours to non-communicable disease prevention, the Declaration catalysed a multisectoral movement to embed physical activity into healthcare, policy, education, and urban design. In this conceptual follow-up article, the GA@PA positions physical activity as the central behavioural target within a broader, integrative precision prevention framework that also considers behavioural, biological, environmental, and social determinants of health. This conceptual framework is intended to support earlier risk detection and intervention. Rather than presenting a fully operationalised system, the article advances a practice-informed conceptual framework outlining how personalised, data-informed strategies could be implemented and scaled across diverse populations, settings, and life stages to improve long-term health outcomes.

This article is intended as a conceptual, implementation-oriented perspective rather than an evidence synthesis, consensus guideline, or systematic review. Accordingly, the article combines current evidence with conceptual interpretation and future-oriented implementation considerations, while explicitly recognising that several components of precision prevention remain emerging and incompletely validated. To clarify the scope, evidentiary basis, and intended contribution of this work, Table 1 summarises key limitations of current physical activity prevention approaches and the rationale for a precision prevention framework. The distinguishing features and intended contribution of the GA@PA framework are summarised in Table 2.

**Table 1:** Current knowledge and contributions of the Global Alliance for the Promotion of Physical Activity (GA@PA) framework

Section	Statement
What is already known	Physical inactivity and sedentary behaviour are major, modifiable drivers of the global burden of non-communicable diseases
	Although genomics, digital technologies, and artificial intelligence are increasingly used for personalised disease management, their proactive application in prevention remains limited. This is primarily due to the scarcity of longitudinal population-level data, challenges in translating research into preventive programs, the complexity of multifactorial interventions, ethical and legal concerns, and economic and incentive structures favouring treatment over prevention [3, 6]
	Generic behavioural advice (e.g., “exercise more”) has limited effectiveness when it is not tailored to individual, social, and environmental contexts
What this paper adds	A narrative, integrative conceptual framework for precision prevention that places physical activity at the centre of personalised health promotion
	The framework aims to support personalised physical activity promotion, behavioural support, and implementation by bringing together stakeholders across multiple sectors, including healthcare, education, urban planning, sport organisations, industry, and community groups
	Emphasis on scalable, low-cost digital and analytical approaches to support individualised prevention across diverse settings
	The framework aligns precision prevention with policy, education, healthcare, sport, and urban planning sectors, positioning the GA@PA as a collaborative implementation platform

**Table 2:** Key messages for clinicians and policymakers

Audience	Key messages
Clinicians	Assess physical inactivity as a vital sign, using feasible and validated tools where available
	Apply individualised physical activity and exercise guidance, informed by the FITT principles and patient context [4, 5]
	Use digital tools (e.g., wearables, mobile platforms) to support motivation, monitoring, and follow-up, where appropriate
	Prioritise early identification and intervention for Exercise Deficiency Syndrome, particularly in higher-risk groups
	Where appropriate, undertake individual risk assessment and clinical evaluation to inform personalised physical activity and exercise recommendations, particularly in individuals with known medical conditions or elevated risk profiles
Policymakers and systems leaders	Incentivise prevention-focused models within healthcare systems, including physical activity promotion
	Support active urban design, including green spaces and an active transport infrastructure
	Strengthen education and training in physical activity, nutrition, and behaviour change across (para-)medical and allied health curricula
	Enable sport and physical activity organisations to promote lifelong participation, aligned with equity and sustainability goals
Strategic perspective	Precision prevention requires a coordinated, cross-sectoral approach to support personalised and population-level physical activity promotion

Across this article, the primary objective of the proposed framework is the promotion of physical activity and prevention of non-communicable disease through personalised prevention strategies. Broader outcomes, including resilience, health span, equity, and societal sustainability, are considered secondary and enabling outcomes rather than equivalent primary endpoints. This paper does not aim to provide a systematic evidence synthesis, clinical guideline, or validated implementation framework. Rather, it proposes a conceptual and implementation-oriented model intended to organise emerging evidence, identify translational opportunities, and define testable priorities for future evaluation.

## ⁠Precision Prevention as a Global Strategic Imperative

Precision prevention is an emerging approach that aims to deliver tailored, personalised preventive interventions by integrating multidimensional data, spanning genetics, epigenetics, environment, education, behaviour, lifestyle, and social determinants of health [6, 7]. While precision medicine primarily focuses on diagnosing and treating disease, based on individual biological, genetic, environmental, and lifestyle characteristics [8], precision prevention broadens the scope. It shifts the focus from treatment to anticipatory health promotion, aiming to maintain well-being and to prevent disease across the lifespan. Crucially, precision prevention addresses both healthy individuals and those at elevated risk, employing interventions that are not only biologically informed, but also context sensitive. This approach contrasts with primordial prevention, which seeks to avert the emergence of risk factors across the entire population. Precision prevention may support more efficient interventions by identifying individuals at heightened risk, whether through behavioural or biomedical markers [6, 9]. Precision prevention strategies alone cannot drive a population-wide shift in risk distribution, because they focus on individuals rather than on the entire population [10]. Instead, precision prevention operates upstream to intervene before clinical disease manifests, while still focusing on those most likely to benefit. Although contextually informed interventions may support broader environments of health, the core function of precision prevention remains the targeted mitigation of individual risk.

A precise intervention is one for which the delivery and design are context-sensitive, informed by detailed, individual or subgroup‑level data, enabling targeted action that is expected to produce differential benefit for the recipients, compared with untargeted or universal interventions. Precision interventions can vary in their degree of tailoring, from stratified subgroup targeting to fully individualised strategies, and are grounded in data that meaningfully stratify risk or intervention response [11].

Many healthcare systems are increasingly challenged by converging pressures, including ageing populations in industrialised nations, the rapid rise of non-communicable diseases (NCDs), widening health inequities, and escalating direct and indirect costs. Without systemic transformation, healthcare expenditures in some countries are projected to exceed 20% of GDP by 2035 [12]. These trends expose the limitations of reactive, treatment-focused care and underscore the urgent need for proactive, preventative, and integrated health systems. A recent Lancet forecasting study further amplifies this urgency, projecting that by 2050, an estimated 746 million children and adolescents (aged 5–24 years) will be living with overweight or obesity, including approximately 360 million living with obesity, compared with 493 million living with overweight or obesity in 2021 [13]. Compounding the obesity crisis is a global decline in physical activity: a pooled analysis of over 5.7 million adults across 163 countries found that insufficient physical activity rose from 23.4% in 2000 to 31.3% in 2022, higher among women (33.8%) than men (28.7%) and increasing in over half of countries worldwide [14]. If current trends persist, the global goal of a 15% reduction by 2030 is unlikely to be met. These findings make clear that chronic conditions like obesity and NCDs are part of a broader ecosystem failure, rooted in behaviours and environments that discourage physical activity. Moreover, over half of the global obesity burden is concentrated in just eight countries [13], and regions including Latin America and Africa remain under-represented in research. Africa faces a dual burden: persistent undernutrition alongside rising obesity. This paradox highlights the necessity for global as well as localised, context-specific investigations that capture region-specific socio-economic, political and individual determinants to inform effective, tailored interventions.

In parallel with the global rise in obesity, the Exercise Deficiency Syndrome (EDS), used here to describe insufficient physical activity to meet minimum health requirements [15], reflects a biologically meaningful exposure, in which lack of physical activity and/or exercise contributes causally to the development of multiple chronic diseases through dysregulation of metabolic, cardiovascular, and inflammatory pathways [16]. EDS is exacerbated by increasingly sedentary lifestyles, including prolonged sitting and other sedentary behaviours (often 8 h or more per day in many adult populations), which are associated with elevated cardiometabolic and all-cause mortality risk [2, 17–19]. Addressing EDS is urgent, as research consistently shows that previously inactive individuals experience disproportionately large health benefits from engaging in physical activity, with particularly large health benefits observed among previously inactive individuals [15, 18]. Educational settings represent an important opportunity to address insufficient physical activity by improving children’s and young people’s awareness and understanding of their movement behaviours. Accelerometer-based findings [20] reveal systematic misperception, pointing to navigable gaps in self-knowledge of physical activity that may influence lifelong behaviour. By supporting teachers and students to address modifiable determinants of physical activity, particularly psychological and social factors such as self-efficacy, perceived behavioural control, and social support, educational settings may help promote sustained participation in physical activity [21]. Physical literacy, defined as the motivation, confidence, physical competence, knowledge and understanding to value and take responsibility for engagement in physical activity throughout the life course, provides a conceptual basis for lifelong participation in physical activity [22].

Environmental changes, including rising global temperatures, are emerging as a critical factor accelerating disease burden and placing additional strain on health systems [23]. Climate change has already led to a measurable increase in the frequency and intensity of extreme heat events, posing disproportionate risks to vulnerable populations, such as older adults and individuals with cardiovascular or respiratory conditions [24, 25]. Within a precision prevention context, building personalised heat resilience is becoming an important emerging frontier. This includes strategies that enhance individual adaptability to thermal stress through improved physical fitness and targeted acclimatisation.

While this conceptual paper primarily focuses on primary prevention, intervening before disease onset, precision prevention strategies are also relevant across the prevention continuum, including secondary prevention through earlier risk detection and tertiary prevention through personalised disease management and rehabilitation [6, 8, 16]. In the context of primary prevention, precision approaches can tailor interventions to promote physical activity and to reduce sedentary behaviour, based on individual risk profiles, preferences, and environments. For instance, personalised physical activity recommendations, digital coaching, and context-aware reminders may support healthy lifestyle changes. Similarly, in secondary prevention, biomarker-based screening and wearable monitoring can aid in the early detection of disease, while in tertiary prevention, digital tools, behavioural support, and personalised care plans may enhance chronic disease management and reduce complications.

**Fig. 1:**
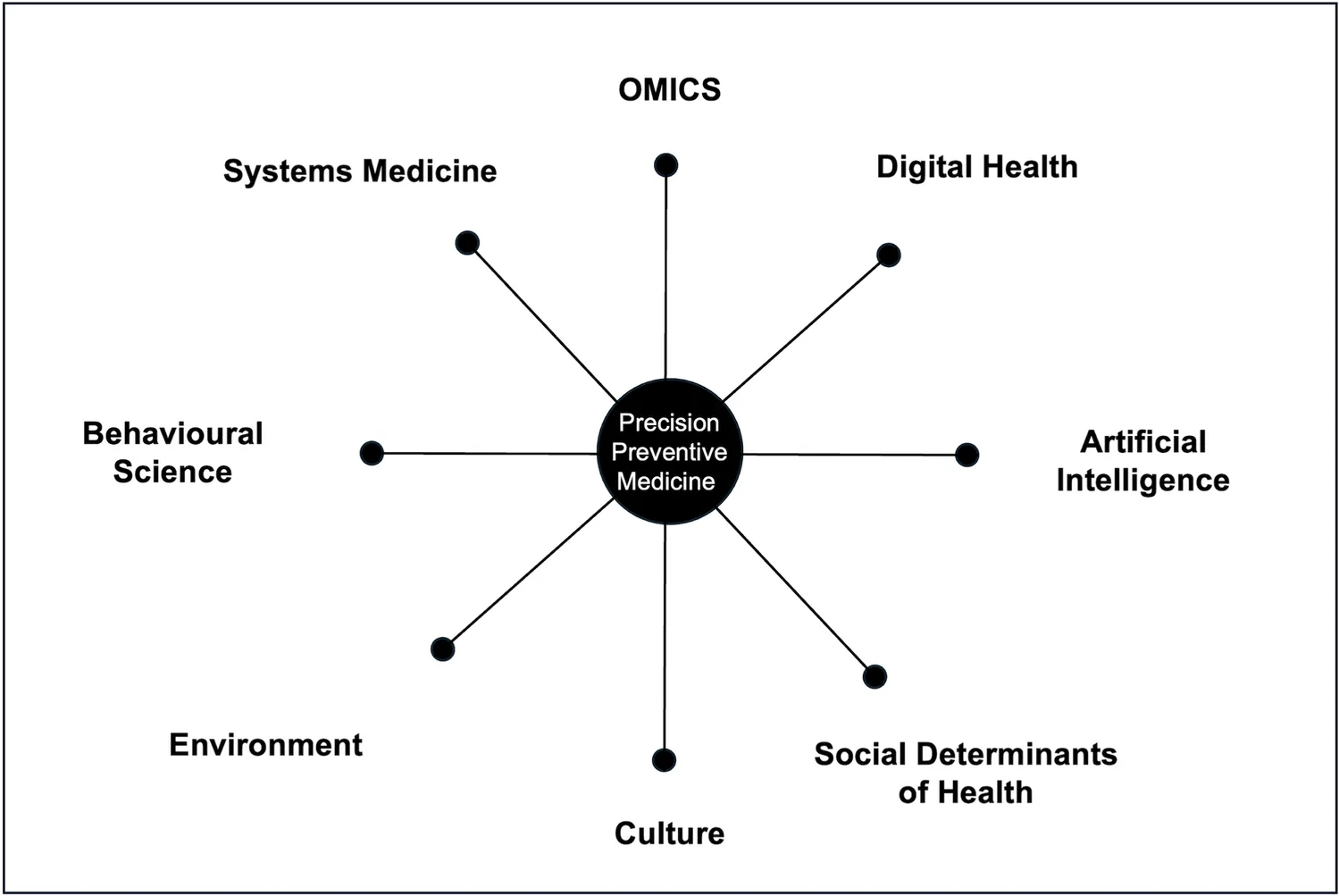
Conceptual representation of the interacting domains, determinants, and enabling fields contributing to precision prevention

Precision prevention has been proposed as a response to several limitations of conventional prevention approaches in traditional healthcare by shifting the focus from episodic, reactive treatment to the continuous optimisation of health and well-being. Notably, physical activity, sedentary behaviour, and physical fitness during childhood are associated with healthier fitness profiles in youth, highlighting the importance of early-life interventions [26]. Precision prevention addresses all levels of intervention, preventing disease onset (primary prevention), identifying early dysfunction (secondary prevention), and supporting chronic disease management (tertiary prevention). By focusing on the early identification of behavioural and physiological risk trajectories across the life course, including behavioural patterns established during childhood and adolescence, precision prevention seeks to support sustained healthy movement behaviours over time [20, 27]. This approach may harness big data analytics, real-time biosensing, and artificial intelligence (AI) to support the identification of individualised risk profiles and potentially inform tailored preventive interventions [8]. Importantly, precision prevention complements population-level strategies by increasing personal relevance and potential impact. Figure 1 illustrates the integrated conceptual framework, showing the convergence of omics, AI, digital health, systems medicine, and behavioural science in informing personalised, proactive care.

Omics refers to the large-scale, systematic study of biological molecules, including genes, transcripts, proteins, metabolites, and related molecular signatures, that contribute to the structure, function, and dynamic regulation of biological systems [28]. Rather than focusing on a single component, omics approaches examine all measurable entities within a specific biological domain, including genes, proteins, metabolites, or transcripts. Key branches include genomics, epigenomics, proteomics, metabolomics, and transcriptomics. In medicine, omics approaches have been proposed as tools that may enable the identification of disease biomarkers, support the development of personalised therapies, and improve predictions of treatment responses. Recent advances in omics have expanded opportunities for earlier disease risk stratification. Genomics, particularly polygenic risk scores (PRS), may support estimation of genetic predisposition years before symptoms emerge. Evidence from UK Biobank studies demonstrates that PRS can interact meaningfully with modifiable behaviours such as physical activity [29, 30]. Even modest increases in physical activity were associated with improved outcomes, particularly among individuals at higher genetic risk [30], reinforcing physical activity as a potent and underutilised intervention target. Beyond genomics, epigenetic and inflammatory markers detectable in childhood have been shown to predict later in life obesity-related disease risk [31–33], while proteomics captures dynamic molecular responses to stress and inflammation, offering functional insight into health resilience [34]. Complementing these biological advances, AI-driven predictive modelling supports patient stratification and adaptive intervention design, enhancing the potential personalisation of preventive strategies [35]. Within this framework, these innovations are presented as enabling tools rather than standalone solutions.

Despite considerable scientific progress, the translational application of many omics-based approaches in prevention remains relatively early, with important challenges persisting regarding validation, implementation, clinical utility, and population-level deployment [6, 36]. Questions remain regarding reproducibility, standardisation, interpretability, cost-effectiveness, and clinical utility outside research settings. Accordingly, while promising, many omics applications should currently be viewed as emerging rather than established preventive tools. Enthusiasm surrounding AI should also be interpreted cautiously. Algorithmic performance is dependent on data quality, representativeness, interpretability, and implementation context, with successful implementation requiring careful consideration of transparency, bias, clinical workflow integration, and real-world deployment challenges [37–39]. Concerns regarding bias, privacy, transparency, and unequal access remain incompletely resolved, particularly in preventive settings where evidence for clinical effectiveness remains heterogeneous [40, 41]

**Fig. 2:**
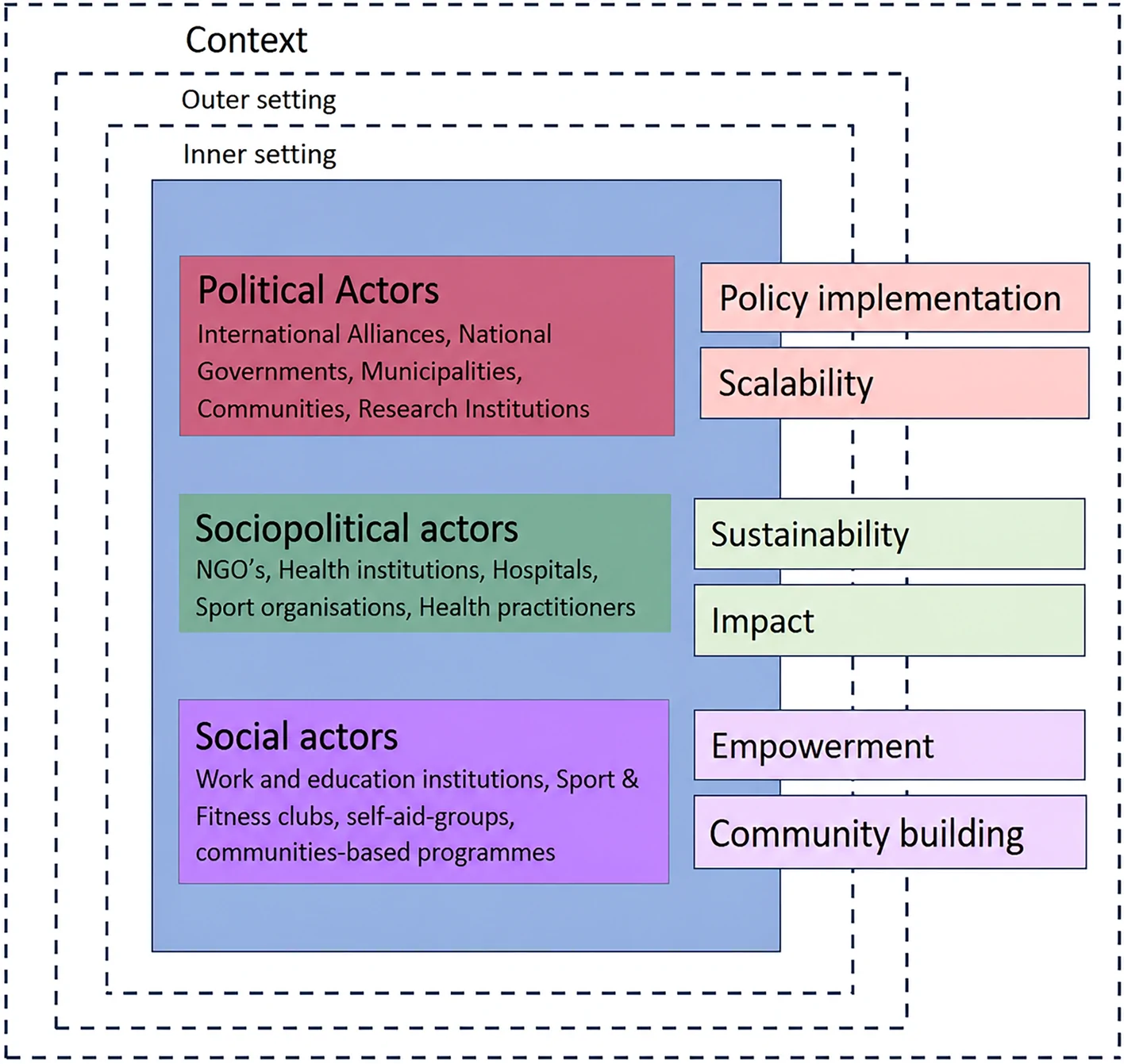
Precision prevention and population-based health promotion and the interactions of various actors at different levels to support and communicate with the target group, adapted conceptually from the framework described in Steinacker et al. [1]

Globally, nations and organisations are beginning to embed elements of precision prevention into policy and practice. China’s Proactive Health strategy [42], the USA’s All of Us Research Programme [43], Europe’s 1+ Million Genomes initiative [44], and the AISEP mission [45] exemplify efforts to move beyond disease-centred health care models towards more personalised, data-informed approaches to prevention and health promotion. Sport has the potential to contribute to global systemic transformation by supporting health promotion, social inclusion, education, and sustainable development when embedded within supportive policy and community frameworks [45–48]. Equally important is the recognition that preventive health initiatives must be globally inclusive, rather than a privilege of high-income populations. Addressing this paradigm shift requires engagement with states, communities, and other key actors. Health is shaped by political, economic, social, and environmental determinants and cannot be understood solely as the product of individual behaviour [49]. A critically informed approach to health research and policy must therefore consider power relations, social inequalities, and epistemic injustice across societies. Safeguarding human dignity remains a foundational principle, and cultural and social dimensions must be meaningfully integrated into prevention strategies. Therefore, stakeholders at all levels (political, socio-political, social, and target groups) need to interact in inner and outer settings, as explained in Fig. 2 [1].

Current international precision health initiatives demonstrate the value of international collaboration, linking individual-level data with population insights. While these current initiatives represent important advances, the GA@PA framework is positioned here as a complementary, implementation-oriented conceptual model. The model moves beyond data collection to emphasise real-world intervention strategies grounded in behavioural science, systems medicine, and environmental design. The Alliance suggests a framework focusing on scalable implementation through tools supporting physical activity and exercise promotion, professional training, community engagement, and policy integration. Bringing together institutions across health, sport, fitness, and policy sectors, this framework positions precision prevention not only as a research agenda but also as a proposed operational conceptual framework for embedding personalised health promotion into everyday settings, with a stated emphasis on equity, cultural adaptation, and global applicability.

Precision prevention should not be viewed as a panacea, but as one component of a broader strategy to address the ever-rising NCD burden. While it offers tools for early risk identification and personalised intervention, it must operate alongside population-wide public health measures. Health is shaped not only by biology, but also by context; i.e., the environments in which people live, move, and work, including social, economic, cultural, and physical determinants that strongly influence health behaviours and outcomes across the life course [16, 50]. Investments in healthy architecture, active mobility infrastructure, green spaces, and inclusive public areas remain essential. Precision approaches must therefore function in tandem with systemic efforts to create environments that support well-being across populations, rather than as isolated, individual-level solutions.

At present, precision prevention should be regarded as an evolving and heterogeneous field rather than a mature, standardised model of care. Many proposed approaches remain variably validated, implementation pathways are inconsistent across settings, and important challenges persist regarding feasibility, equity, interoperability, cost, and evidentiary maturity. Accordingly, the concepts presented here should be interpreted as implementation-oriented directions for evaluation rather than definitive solutions.

### Recommendations and Pathways Forward

To realise the potential of precision prevention as a global health strategy, several key actions warrant prioritisation:

1. **Integrate multidimensional data:** Health systems should adopt frameworks that combine genomic, behavioural, environmental, and social data to inform personalised interventions, while investing in an interoperable and ethically governed data infrastructure.
2. **Prioritise physical activity and exercise:** Addressing Exercise Deficiency Syndrome (EDS) through school-based programmes, workplace policies, and community initiatives should remain central, with activity promotion tailored to individual risk profiles and contexts.
3. **Strengthen early-life interventions:** Evidence supports targeting physical fitness and behavioural risk trajectories from early childhood. Embedding physical literacy, alongside health education, within school curricula may support long-term engagement.
4. **Promote global equity in research and implementation:** Research efforts should expand to under-represented regions, including Africa and Latin America, with localised, context-sensitive study designs.
5. **Build resilience to climate heat stress:** Personalised strategies to enhance heat resilience, including fitness and acclimatisation protocols, should be integrated where relevant to address climate-related health risks.
6. **Align with public health infrastructure:** Precision prevention must complement, not replace, population-wide approaches, including investments in active transport, green spaces, and equitable access to cure and care.
7. **Promote sport-for-all initiatives:** Sport organisations should support inclusive sport-for-all programmes. Initiatives such as the IOC’s Olympism365 may provide examples of scalable implementation pathways.
8. **Foster cross-sector collaboration:** Sustained cooperation across health, sport, education, and policy sectors is essential to implement scalable, inclusive, and culturally adaptive prevention strategies.

## Advancing Scientific Foundations for Precision Prevention

While the value of proactive, preventive medicine is increasingly recognised, its implementation remains nascent, hampered by a fragmented, discipline-siloed evidence base that lacks the integrative structure needed for global adoption. To realise its full potential, precision prevention must evolve from conceptual aspiration into a robust, interdisciplinary conceptual framework grounded in molecular biology, systems medicine, behavioural science, and environmental health, all applying systems thinking. Such a foundation must honour both biological individuality and the common patterns of health and disease that emerge across populations, cultures, and contexts [6]. At its core lies a deceptively simple, but still unresolved question: what does it mean to be healthy? Health is not merely the absence of pathology, but a dynamic capacity for adaptation, recovery, and resilience, best understood through how individuals respond to stress and maintain function across changing demands, with disease often reflecting maladaptive responses to an individual’s social, physical, economic, cultural, and biological environment.

Conceptually, the proposed framework aligns with broader theories of health that emphasise interactions between biological, behavioural, psychological, social, and environmental influences [51]. Salutogenic perspectives emphasise the promotion of resources that support health, resilience, and adaptive capacity rather than focusing exclusively on disease risk [52]. Biopsychosocial and socioecological models similarly conceptualise health as emerging through dynamic interactions across multiple levels of influence, ranging from biology and behaviour to social context and environmental opportunity [53]. Within this context, physical activity may function as a central behavioural mediator linking biological adaptation, behavioural regulation, social participation, and environmental opportunity [54]. Precision prevention is therefore conceptualised not as a replacement for existing health frameworks, but rather as a potentially complementary implementation-oriented model through which these principles may be operationalised.

Environmental exposures, ranging from poor sleep and nutrient deprivation to climate extremes, are stressors that increasingly challenge this adaptive capacity. For example, the response to compound heatwaves, including prolonged periods of elevated night-time temperatures, has been reported to be associated with a near-linear increase in mortality risk, particularly among individuals with pre-existing cardiovascular conditions [55, 56]. Such findings underscore the need for personalised prevention strategies that also enhance resilience to environmental stress, whether by improving baseline fitness, optimising hydration and thermoregulation, or leveraging targeted heat acclimation protocols that build on individual physiological adaptability [57].

Shaping personalised prevention strategies may require high-resolution, longitudinal data that capture physiological, metabolic, epigenetic, psychological, and behavioural signals across time. These must be context-sensitive, capturing environmental exposures, socio-economic conditions, cultural practices, and lived experience. However, current research often neglects the critical transitions that shape health across the life course. We lack predictive models for how stressors, whether acute or chronic, physical or psychosocial, translate into either physiological deterioration or adaptive resilience. Exposures such as physical inactivity, nutrient deprivation, chronic inflammation, poor sleep, and environmental pollutants activate complex biological cascades that remain poorly understood. Furthermore, oral health should not be neglected, as longitudinal evidence indicates that metabolic risk factors, such as obesity, are associated with adverse oral and dental outcomes during adolescence [58], and poor oral health is itself linked to chronic disease risk in later life [59]. In addition to systemic benefits, physical activity has been associated with improved oral and dental health outcomes in observational evidence and sport-health literature [60], potentially via inflammatory, metabolic, and behavioural pathways [17]. Crucially, the impact of these stressors is not uniform: it is shaped by social determinants, such as educational attainment, income level, and psychosocial coping capacity, including factors like marital status and social support. These modifiers can either buffer vulnerability or amplify risk, influencing whether resilience is maintained or lost. Identifying the tipping points where adaptation gives way to dysfunction, while accounting for these contextual influences, is essential for the early detection and targeted prevention that precision prevention aspires to deliver.

Exceptional individuals like elite athletes, centenarians, and so-called “at-risk populations”, those with unusually high or low resilience, longevity, or recovery capacity, offer valuable insights into mechanisms of health preservation. Studies of twins, family clusters, and rare phenotypes can reveal conserved pathways and novel genes related to health span, immunity, metabolic regulation, low-grade inflammation or cognitive performance. Such approaches must be studied and interpreted in conjunction with social and cultural factors. Traditions, community dynamics, beliefs and personal motivations may, in some circumstances, have a significant influence on behaviour and, consequently, on potential interventions.

Evolutionary medicine provides a useful framework for understanding how mismatches between contemporary environments and human biology may contribute to the growing burden of chronic disease. Central to this perspective is the mismatch hypothesis: the notion that human physiology, shaped over millennia for environments characterised by regular physical activity, intermittent food supply, and rich social engagement, is poorly adapted to today’s sedentary, energy-dense, and psychologically fragmented lifestyles. This biological environmental mismatch underpins the rise of obesity, type 2 diabetes, cardiovascular disease, and mental health disorders. This mismatch suggests that contemporary environments may increasingly challenge physiological systems that evolved under substantially different behavioural and environmental conditions [60]. While numerous biological pathways have been described through which physical activity promotes health, the mechanistic understanding of sedentary behaviour and physical inactivity continues to evolve [61]. Precision prevention aims to bridge this gap by leveraging advances in biological, behavioural, and data sciences to support healthier adaptation to rapidly changing environments. Comparative biology, including cross-species research, may further enrich this perspective by challenging overly reductionist models of disease and ageing.

At the systems level, health status emerges from the dynamic interplay of factors operating across multiple scales, from molecular and physiological processes to cognitive states and the surrounding social and physical environment. Capturing these interconnections requires advanced modelling approaches, capable of reflecting complex, non-linear dynamics across time and context. A diverse range of methodologies, including systems biology, digital twins, multi-scale simulations, causal inference frameworks, and AI and machine-learning algorithms can help map these relationships and simulate personalised health trajectories [37–39]. Consistent with Rose’s population health framework, personalised prevention should complement, rather than replace, population-wide prevention strategies aimed at shifting disease risk across the whole population [62]. When integrated with wearable or implantable sensors, such models enable real-time, functional health monitoring and may pave the way for anticipatory, individualised interventions [63]. For instance, wearable technologies and digital-twin approaches are increasingly being explored for cardiovascular risk prediction, assessment, and monitoring, providing data to support early detection, clinical decision-making, and personalised treatment [64, 65]. In the field of sport, real-time monitoring of core temperature via ingestible or skin-mounted sensors is increasingly used to protect athletes competing in extreme heat, helping prevent heat stroke and performance collapse through personalised risk feedback [66]. Advances in implementation science, digital health technologies, data integration, and artificial intelligence may increasingly support elements of this vision, although important scientific, ethical, and implementation challenges remain. The translational maturity of precision prevention approaches varies considerably across domains, with some applications already entering practice while others remain largely investigational. Considerable challenges remain regarding interoperability, feasibility, implementation cost, user engagement, privacy, and equitable access across diverse settings. Accordingly, the framework outlined here should be interpreted as an evolving conceptual direction rather than a mature implementation standard.

### Recommendations and Pathways Forward

To advance the scientific foundations of precision prevention, the following priorities should guide future research and implementation:

1. **Establish interdisciplinary frameworks:** Integrate molecular biology, behavioural science, systems medicine, and environmental health into a unified model. This requires breaking down academic silos and promoting collaboration across disciplines and sectors.
2. **Redefine health dynamically:** Move beyond static definitions of health as disease absence. Emphasise resilience, adaptability, and recovery capacity, with particular focus on how individuals respond to stressors across biological, social, and environmental domains.
3. **Capture longitudinal, context-aware data:** Invest in high-resolution, life-course data collection, including physiological, psychological, metabolic, and epigenetic signals, while accounting for lived experience, cultural norms, and socio-economic conditions.
4. **Leverage insights from exceptional populations:** Study elite athletes, centenarians, and genetically or socially unique groups to uncover mechanisms of resilience, longevity, and adaptation, integrating biological and psychosocial determinants.
5. **Embed evolutionary perspectives:** Address the mismatch between evolved human physiology and modern environments by promoting lifestyles that align with our biological design, particularly through regular physical activity, balanced nutrition, and social connectivity.
6. **Apply advanced modelling and AI:** Develop and validate personalised health models using AI, causal inference, digital twins, and wearable sensors to enable anticipatory, individualised interventions.
7. **Invest in infrastructure and ethics:** Prioritise interoperable digital platforms, sensor technologies, and ethical governance to ensure equitable, secure, and actionable precision health applications.

## Technological Infrastructure for Precision Prevention

The realisation of precision prevention as a practice-oriented health framework depends not only on scientific insight, but also on the development of enabling technologies (Table 3). These technologies must continuously capture, process, and interpret complex health data in ways that are meaningful to individuals, clinicians, healthcare systems, and prevention. They must also be sensitive to the temporal, spatial, and contextual nature of health and adaptable across diverse cultural and infrastructural environments, including remote areas and populations with limited mobility or healthcare access [67]. Beyond data handling, such technologies may also support tailored health education and behaviour change strategies, empowering individuals and communities to make informed decisions that could contribute to reducing mortality associated with physical inactivity.

**Table 3:** Examples of key technologies enabling precision prevention

Technology	Function	Current applications	Challenges
Biosensors	Real-time health monitoring	Dehydration, glucose monitoring	Standardisation, affordability
Digital twins	Simulate health scenarios	Cardiovascular risk prediction	Data integration, privacy
AI analytics	Predictive and personalised insights	Disease modelling, diagnostics	Handling complexity, bias and privacy
Wearables	Track physical and metabolic data	Activity, sleep, stress tracking	Interoperability, user adherence

At the heart of this technological transformation are integrated biosensing systems. Wearable, implantable, and ambient sensors are redefining real-time health monitoring by dynamically tracking physiological, metabolic, and behavioural states in everyday environments. Recent advances in nanomaterials, optoelectronics, and artificial intelligence have enabled these devices to non-invasively detect key indicators, such as hydration status, glycaemic variability, microvascular reactivity, and emotional stress through proxies such as skin conductance and heart rate variability [68, 69]. The development of flexible, skin-like materials supports continuous, long-term use, while embedded algorithms allow for real-time interpretation and context-aware personalised feedback. Recent breakthroughs in wearable sensor technologies further extend this potential. For example, sensors integrated into clothing can support continuous physiological monitoring, contributing to unobtrusive health tracking in everyday settings [70]. These innovations open new opportunities for unobtrusive, continuous health tracking in everyday settings. However, several challenges remain. Ensuring accuracy and reliability in real-world conditions, addressing affordability, achieving data interoperability, and ensuring equitable access across diverse global populations remain essential prerequisites for translating these technologies into scalable and inclusive healthcare solutions.

Despite their promise, several practical barriers limit the translation of precision medicine, precision prevention and AI technologies into routine healthcare. The GA@PA framework proposes evidence-based and collaborative solutions to address these challenges [1]. Accuracy and reliability remain critical concerns, as models validated in controlled settings may underperform in real-world clinical environments. These limitations can be addressed through prospective multicentred validation, continuous performance monitoring, and rigorous quality control frameworks [71]. Affordability and scalability pose major challenges, particularly in low-resource settings. These barriers may be mitigated through tiered implementation strategies, cloud-based infrastructure, and alignment with value-based healthcare models [37]. Data interoperability is essential to integrate patient information across systems. This can be achieved through the adoption of common data standards, bridging middleware (software enabling interoperability between otherwise incompatible health information systems), and collaborative data-sharing initiatives [72]. Equitable access must be ensured to avoid exacerbating existing health disparities. This requires diverse representation in training datasets, community-based deployment programmes, and systematic monitoring of outcomes across demographic groups [71, 73]. Addressing these barriers pragmatically is essential if these technologies are to achieve scalable, inclusive, and sustainable implementation in clinical practice.

Digital twin technology is emerging as a potentially enabling model for individualised health forecasting [75] and personalised medicine, enabling virtual representations of biological systems that can be used to simulate disease trajectories and intervention responses [75]. These representations can be used to simulate the potential outcomes of personalised interventions before they are applied in real life, supporting predictive testing and decision-making [64]. As these models mature, they may become increasingly relevant not only to clinical workflows, but also to proactive, self-directed health optimisation [63]. Precision prevention strategies, particularly those involving physical activity, sleep, and nutrition, are associated with reductions in the risk of chronic disease, frailty, and all-cause mortality. It is important to analyse mortality and morbidity risk based on individualised health data by AI methods rather than by classical group statistics which have their limits when considering small effect sizes in prevention. AI is therefore a tool for analytical approaches rather than referring only to big language systems. AI can be used in closed logical systems from small to big data. This may enable better risk prediction for prevention strategies and reinforce the value of early intervention which favours systems-thinking. Health is an emergent property arising from biological, psychological, social, and contextual interactions. Accordingly, analytical methods must move beyond conventional statistics and linear assumptions toward advanced modelling tools capable of capturing dynamic interactions, modelling individual health trajectories, and accommodating real-world variability [76]. This integration positions digital twins as a potentially transformative, though still developing, asset within precision prevention, enabling personalised cure, care and prevention that is anticipatory, adaptive, and context aware.

While the practical deployment of digital twins for individualised risk calculation remains in its early stages, proof-of-concept studies have demonstrated promising applications. AI-driven avatars have been piloted in modelling metabolic, cardiovascular, and cognitive health trajectories by integrating biosensor data with predictive algorithms [77, 78]. These systems can simulate intervention outcomes and provide real-time risk estimation, laying the groundwork for highly personalised and anticipatory health strategies. However, large-scale validation and clinical integration are still required to ensure data quality, model transparency, and robustness across diverse populations and environments. Complex and variable environmental contexts remain insufficiently captured by current digital twin architectures, necessitating further methodological development and evaluation [46].

E-health strategies, particularly mobile health (m-health), can expand the reach of preventive interventions by leveraging widely available technologies, such as smartphones and basic mobile phones, across socioeconomic levels and age groups. Through formats such as short videos, daily messages, tailored recommendations, interactive infographics, and educational capsules, these interventions can deliver personalised guidance and home-based physical activity and exercise routines using readily available resources. Their ease of access and application has been shown to support engagement, including in remote or underserved communities. A recent umbrella review and meta-analysis encompassing 507 randomised controlled trials with over 206,000 participants reported that e- and m-health interventions significantly increased physical activity levels, with an average increase of 1329 steps per day and 55 min of moderate-to-vigorous physical activity per week [79]. These findings highlight the capacity of digital health tools to promote physical activity across diverse populations and settings.

While advanced technologies such as biosensors, AI, and digital twins hold substantial promise, their implementation must be context sensitive. In low-resource settings, simplified approaches, such as mobile-based health tracking, community-led monitoring, and locally adapted risk assessment tools, can offer immediate benefits. These strategies leverage existing infrastructure and cultural practices to deliver personalised prevention in accessible formats. Within the GA@PA framework, tiered implementation strategies are emphasised, ensuring that where high-technology solutions are not yet feasible, core principles of precision prevention, personalisation, proactivity, and empowerment remain actionable and equitable. Collectively, these approaches illustrate how digital health tools, when integrated thoughtfully, can contribute to preventive care without exacerbating inequities.

Just as the World Health Organization (WHO) underscores the importance of integrating traditional medicine into national health strategies, particularly in under-resourced settings [80], there is growing interest in complementing pharmacological care with culturally grounded, non-pharmacological approaches. When scientifically validated and digitally supported, traditional practices may enhance prevention and behaviour change strategies that are cost-effective and locally acceptable. Nutritional and plant-based interventions, for example, have been proposed as adjuncts to conventional treatments, particularly in the management of chronic low-grade inflammation [81, 82]. Advancing such integration will require coordinated efforts to address challenges, including the standardisation of biosensing technologies, harmonisation of data formats, and development of shared validation protocols [6].

Equity and accessibility must guide the design and deployment of precision health technologies. Devices should be non-invasive, energy-efficient, affordable, culturally appropriate, and usable by individuals without clinical training. Evidence from human-centred design and digital health equity research indicates that low-complexity interfaces and contextual sensitivity are associated with higher adherence and engagement, particularly in underserved populations [68, 83]. However, even well-designed tools are insufficient in the absence of supportive environments. For example, accelerometry-based interventions for physical activity have demonstrated limited effectiveness in areas lacking safe outdoor spaces or walkable infrastructure [84]. Similarly, nutritional tracking applications depend on access to affordable, healthy food, and stress-monitoring wearables are most effective when paired with behavioural health support. Public health systems, urban design, and workplace policy could be improved by digital tools to ensure that measurement translates into sustained behavioural change [68].

In summary, the future of precision prevention depends on the convergence of biomedical engineering, computational modelling, the built environment, systems theory, behavioural science, and cultural and contextual understanding. This convergence represents enabling infrastructure rather than a standalone solution, and its success will depend on thoughtful implementation, evaluation, and governance.

### Recommendations and Pathways Forward

To realise the promise of precision prevention, technological infrastructure must evolve toward greater accessibility, integration, and equity. The following actions are prioritised:

1. **Develop interoperable and scalable technologies:** Standardise biosensor platforms, digital twin frameworks, and AI models to support accurate, reliable, and cross-compatible health monitoring, while prioritising affordability and contextual adaptability.
2. **Promote human-centred and inclusive design:** Ensure technologies are usable by non-experts and effective across cultural and socioeconomic settings, leveraging familiar devices such as mobile phones.
3. **Validate and integrate digital twins:** Support rigorous clinical and population-level validation of digital avatars for risk prediction and intervention modelling, while addressing data quality, privacy, and contextual sensitivity.
4. **Expand mobile health (m-health) interventions:** Use SMS, video, and interactive content to deliver culturally relevant behaviour change strategies, particularly in low-resource settings.
5. **Embed digital tools in supportive environments:** Pair digital interventions with access to safe physical spaces, healthy food, and behavioural support to enable sustained change.
6. **Integrate traditional and digital approaches:** Where scientifically validated, traditional practices can be incorporated alongside digital tools to enhance engagement and adherence.
7. **Ensure ethical and equitable implementation:** Embed digital health ethics, transparency, and equity considerations into technology design, policy, and deployment.

## Intervention Strategies for Precision Prevention

Early intervention lies at the heart of precision prevention, where conceptual frameworks are translated into practice, data inform both prevention and care, and science interrogates human behaviour in complex, real-world environments. The challenge is to move beyond generic lifestyle advice and reactive treatments toward interventions tailored to individual biology, behaviour, context, and life stage (Table 4). The foundational premise is that human health can be supported and enhanced through personalised combinations of rest, physical activity and exercise, nutrition, cognitive stimulation, environmental alignment, and social connection. Precision prevention interventions are inherently multidimensional, integrating key lifestyle and environmental factors to support personalised health outcomes such as mental health, sleep, physical activity and nutrition. These elements modulate inflammation, metabolism, neural plasticity, and cellular repair, reflecting the principle that biological systems adapt to repeated behavioural and environmental exposures [85]. For instance, low-grade inflammation disrupts structural plasticity in the brain, highlighting the importance of targeting inflammatory pathways to preserve cognitive function through molecular and cellular mechanisms [86, 87].

**Table 4:** Life-stage specific intervention strategies (not limited)

Life stage	Key risks	Recommended interventions	Expected outcomes
Toddlers	Nutritional deficiencies, delayed milestones, impaired motoric activity	Balanced diet, early stimulation, active playing	Improved development, growth, and immune health
Childhood	Developmental issues, obesity, osteoporosis prevention, impaired learning, impaired motoric learning	Nutrition, physical activity within school and during free time, active playing, low screen use	Healthy growth, disease and obesity prevention, resilience, bone health
Adulthood	Metabolic syndrome, stress, cardiovascular disease	Precision exercise, mental health support	Disease prevention, productivity, heart health, metabolic health
Older Adults	Cognitive decline, frailty	Sleep optimisation, critical nutrients, strength training, mobility programmes	Brain and metabolic health, physical independence

Physical activity and exercise are the cornerstone of this model. Precisely calibrated physical activity and exercise can function as both prevention and therapy, echoing Hippocrates’ observation that balanced nourishment and exercise are central to health. This principle is now supported by evidence demonstrating that exercise has been effectively prescribed across at least 26 chronic diseases [88]. However, not all physical activity and exercise interventions are equally effective, as outcomes depend on biological variability, individual motivation, and environmental context. Accordingly, integrating exercise science with behavioural psychology and urban design is essential to enhance effectiveness, sustainability, and equity.

The European Federation of Sports Medicine Associations (EFSMA) ten-pillar framework [15] is presented here as an illustrative implementation example demonstrating how principles of precision prevention may be translated into operational public health and sports medicine structures. The model highlights ten inter-related domains: the consequences of change through exercise; the interaction between genetics and environment; the health effects of sedentary lifestyles; reasons for physical inactivity and pathways to becoming physically active; strategies for promoting physical activity through personal motivation; use of the Motivation Pyramid to support behavioural change; general advice for effective intervention strategies; identification of barriers to exercise and methods to overcome them; the role of wearable technologies and other novel motivational tools; and the benefits of physical activity counselling in clinical practice. Together, these domains provide a structured and practical approach for implementing personalised physical activity and exercise interventions across healthcare and community settings.

**Fig. 3:**
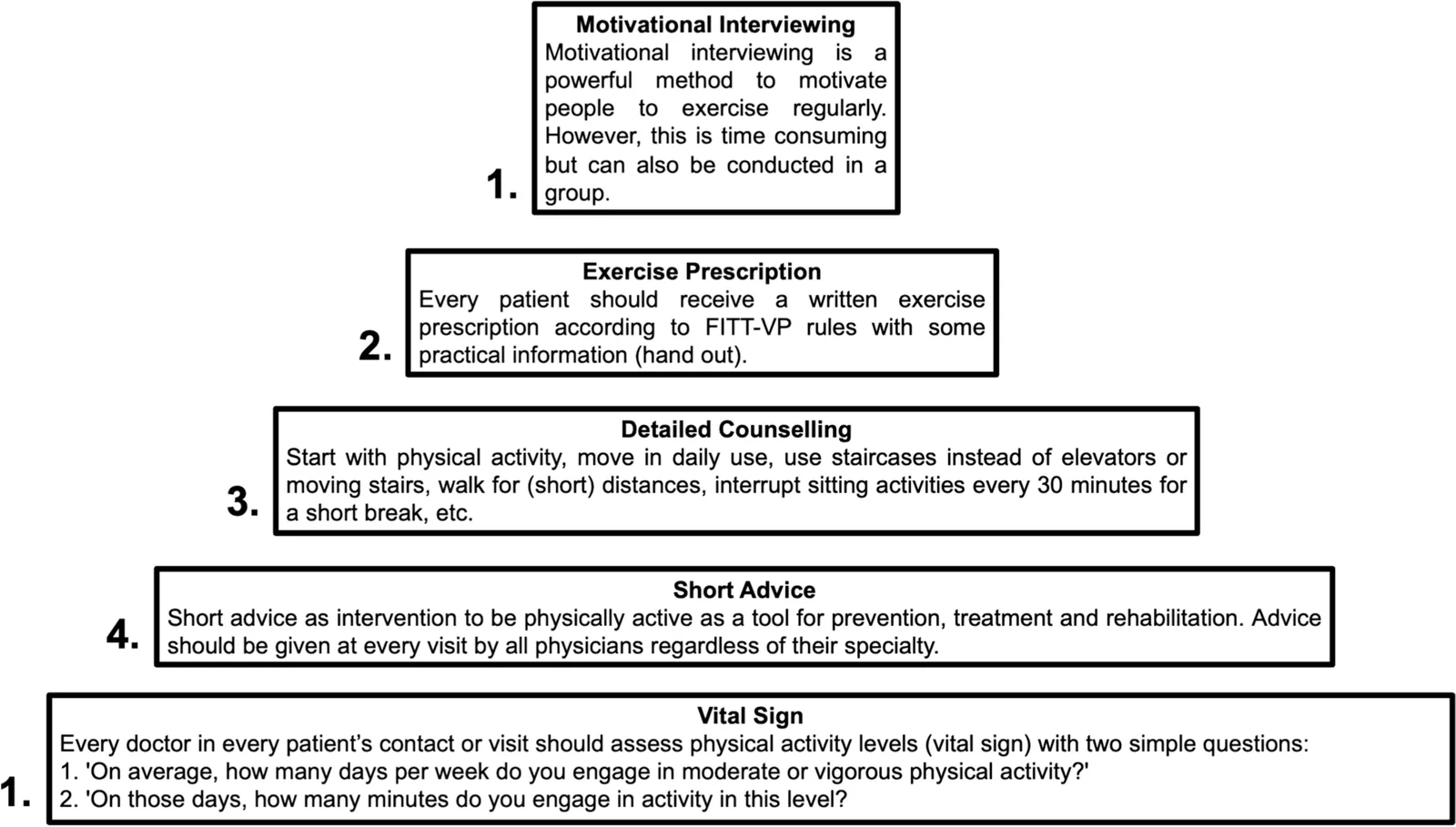
Motivation Pyramid illustrating the hierarchical organisation of motivational influences on behaviour and physical activity participation. The model highlights how foundational motivational factors may support sustained engagement in physical activity and inform personalised prevention strategies within the proposed Global Alliance framework. Adapted from Löllgen et al. [15]

The model introduces tools such as the Motivation Pyramid (Fig. 3) and the FITT principle (Frequency, Intensity, Time, and Type) to support physical activity and exercise prescription and behavioural adherence. These tools align with the core tenets of precision prevention, personalisation and contextualisation of behaviour change strategies. In clinical settings, Exercise Prescription for Health based on the 10-Pillar Model proposes one example of a structured implementation pathway. Physicians are encouraged to assess physical activity and exercise levels as a vital sign and to provide written, individualised prescriptions [19]. Consistent with guidance from the American College of Sports Medicine, (para)medical practitioners across specialties are encouraged to assess physical activity as a vital sign, helping to normalise exercise as a clinical standard [89]. The 10-Pillar Model also underscores the importance of medical education reform, calling for greater integration of sports and exercise science and medicine into undergraduate and postgraduate curricula [15, 90]. These elements provide practical pathways to scale physical activity and exercise interventions, address barriers to engagement, leverage digital tools for motivation, and support longer-term adherence. The EFSMA10-Pillar Model is included not as a definitive model, but as one potential implementation pathway aligned with the broader conceptual aims of precision prevention.

**Fig. 4:**
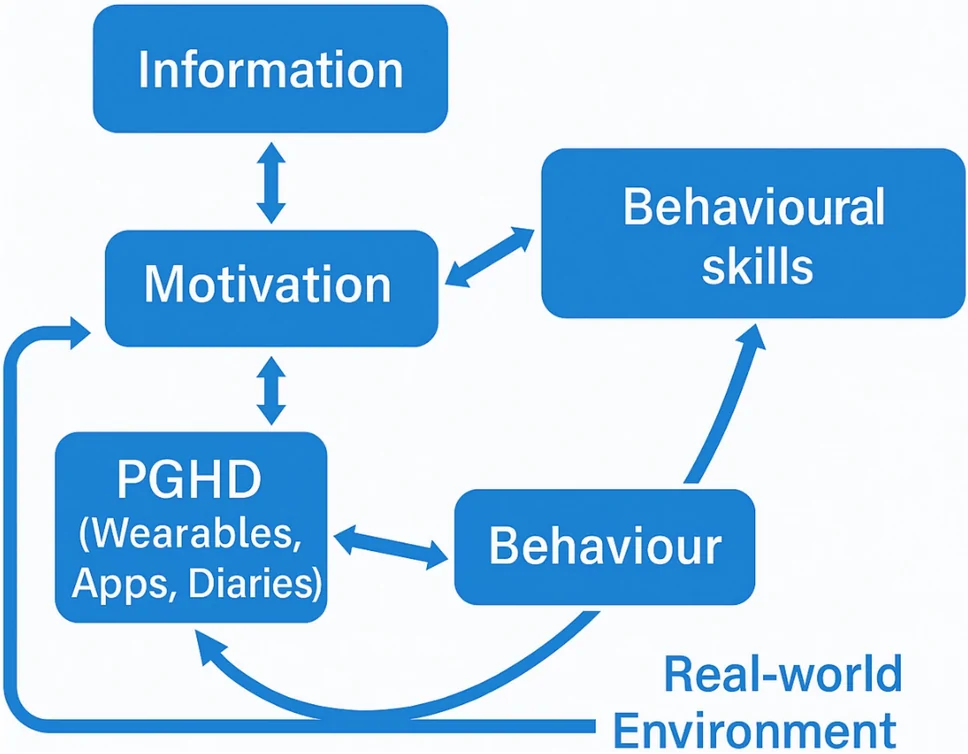
Adapted Information–Motivation–Behavioural Skills (IMB) model incorporating Person-Generated Health Data (PGHD). The model illustrates how information, motivation, and behavioural skills interact to modify behaviour, while also integrating bidirectional feedback loops from PGHD (e.g., wearables, apps, diaries) and real-world environmental influences. Based on the conceptual framework proposed by Fisher et al. [91]

Alongside the 10-Pillar Model and the Motivation Pyramid, the adapted Information–Motivation–Behavioural Skills (IMB) model (Fig. 4) proposes a foundational behavioural framework. Its application is enhanced through the integration of person-generated health data (PGHD), including physical activity, sleep, and physiological metrics from wearables and mobile applications. While clinical encounters can deliver information and motivation, behaviour change occurs in real-world settings where individuals apply behavioural skills and generate continuous data streams.

PGHD functions as a feedback mechanism, enabling personalised reinforcement, early identification of adherence barriers, and real-time adjustment of goals. Through this process, information becomes personalised, motivation is sustained through feedback, and behavioural skills are reinforced through self-monitoring and adaptive goal setting. Within this conceptual model, integration of PGHD strengthens the linkage between episodic clinical care and continuous behaviour support [91].

Reducing sedentary time, particularly prolonged sitting, has been independently associated with improved metabolic, cardiovascular, and mental health outcomes, even among individuals meeting physical activity guidelines [18, 92]. Explicit inclusion of sedentary behaviour strategies would enhance the relevance and scalability of intervention frameworks for diverse populations.

Nutrition represents another core intervention domain. Evidence suggests that physical activity and diet are complementary health behaviours, and that integrated interventions targeting both may be more effective than addressing either in isolation [92]. However, gaps in nutrition training among (para)medical professionals persist and warrant attention through expanded education [21]. Precision nutrition focuses on optimising interactions between diet, metabolism, and gene expression. Tailored dietary strategies can modulate immunity, oxidative stress, and metabolic regulation, influencing pathways related to neurodegeneration and ageing [82, 93–95]. Real-time tracking using digital tools now informs personalised dietary guidance [96]. Cultural food systems, fasting traditions, socio-economic constraints, and adverse food reactions must also be considered within integrative models [97].

Sleep and circadian regulation are foundational to health. Precision sleep interventions aim to restore biological rhythms through behavioural modification, light exposure, temperature control, and acoustic stimulation. Advances in wearable monitoring allow earlier detection of sleep disruption, mood disorders, and cognitive decline. Circadian alignment plays a critical role in brain function and overall health by synchronising biological rhythms with environmental and cultural cues [98, 99].

Well-being in all dimensions (mental, health, psyche) is central to precision prevention because human well-being relates to physical health and behaviour. Emerging technologies enable monitoring health and mental states through biometric feedback and affective computing which should support the individual. Wearable neuro-technologies, including transcranial direct current stimulation (tDCS), have been promoted for supporting emotional regulation and cognitive control [100]. Recent developments in closed-loop systems allow adaptive responses to users’ emotional states in real-world contexts [101, 102]. These approaches remain adjunctive and require the utmost careful evaluation, with personalisation, cultural sensitivity, and ethical oversight remaining essential.

The environment, including urban design, green and blue spaces, housing quality, transport networks, and community infrastructure, acts as an active enabler of health behaviours. These features reduce barriers to movement, support sleep quality, and shape dietary choices. Precision prevention must therefore integrate behavioural enablers such as social prescribing, architectural audits, and policy interventions that foster health-promoting environments [103].

Addressing upstream determinants such as poverty, education, and inequality remains critical, as these factors shape context and opportunities for sustained behaviour change [104]. Interventions must also be life-stage sensitive. In this context, expanding the traditional sports medicine pre-participation evaluation (PPE) beyond athletic populations may provide an entry point for preventive counselling in youth and young adults [103]. Youth engagement strategies should emphasise co-design and participatory approaches.

Delivering this conceptual framework and vision requires a professional ecosystem that includes clinicians and allied health professionals trained in behavioural coaching, systems thinking, and cultural competence. The American Heart Association’s “Life’s Essential 8” framework [104] proposes a useful example of how core lifestyle and cardiovascular risk factors can be integrated into precision prevention strategies. These include smoking status, physical activity, body weight, diet quality, sleep duration, blood pressure, blood lipids, and blood glucose. Within the American Heart Association’s Life’s Essential 8 cardiovascular health framework, these domains may provide a practical foundation for cardiovascular health assessment and prevention, helping to guide risk assessment, behavioural counselling, monitoring, and preventive interventions across diverse populations [104]. Combining behavioural factors such as physical activity, sleep, and diet with physiological markers such as blood pressure, lipid levels, and glycaemic control supports a more comprehensive and context-sensitive approach to health promotion and chronic disease prevention [104]. Early identification and quantification of cardiovascular risk factors remain central to cardiovascular disease prevention and the promotion of optimal cardiovascular health [104]. Scalable impact will depend on continuous, community-integrated care supported by aligned incentives and sustained policy commitment.

While general dietary guidance remains important, evidence supporting specific dietary patterns, such as the Mediterranean diet, suggests that more explicit recommendations may offer added benefit [105, 106]. Similarly, BMI (body mass index) has recognised limitations, particularly in physically active individuals, and waist circumference may provide additional predictive value when combined with activity measures [107]. Furthermore, reliance on biomarkers, such as HbA1c, highlights the need for globally accessible and standardised indicators, including emerging continuous glucose monitoring approaches.

In summary, precision prevention interventions must be multidimensional, scientifically grounded, culturally informed, and embedded within real-world contexts. This section presents an implementation-oriented synthesis rather than prescriptive clinical guidelines, emphasising adaptability, evaluation, and equity.

### Recommendations and Pathways Forward

Precision prevention interventions must be holistic, tailored, and life-stage specific. To achieve scalable impact, the following priorities are proposed:

1. **Adopt structured physical activity frameworks:** Integrate the principles of the 10-Pillar Model as an implementation tool for personalised physical activity and exercise promotion, with ongoing evaluation across settings.
2. **Prescribe physical activity as a clinical standard:** Encourage routine assessment of physical activity as a vital sign and support clinicians and allied professionals with evidence-based tools.
3. **Expand medical education:** Strengthen training in exercise science, nutrition, behavioural health, and motivational interviewing.
4. **Prioritise multidimensional, life-course approaches:** Integrate physical activity, nutrition, sleep, mental health, social connection, and environmental design.
5. **Enhance nutrition literacy:** Improve training in precision nutrition and culturally responsive dietary counselling.
6. **Address mental and circadian health:** Incorporate personalised sleep and mental well-being strategies supported by digital tools.
7. **Address environmental and socioeconomic barriers:** Embed interventions within health-promoting environments and address structural inequities through policy and community co-design.

## Necessary Next Steps

As precision prevention evolves from concept to operational reality, structured, scalable, and collaborative action becomes essential. Scientific breakthroughs alone are insufficient; they must be integrated into cure and care systems, education, industry, and governance. Accordingly, precision prevention is framed here not as a single intervention, but rather as a coordinated systems-level strategy. A global agenda is needed to position precision prevention as a pillar of sustainable health and societal resilience [6]. A critical first step is establishing an international research initiative prioritising interdisciplinary, systems-level health enhancement studies. Large-scale, longitudinal studies are required to harmonise diverse datasets, define dynamic health baselines, and map ageing and disease trajectories under varying contextual conditions [26]. Emphasis should be placed on populations at the extremes of resilience and vulnerability, including outlier populations demonstrating exceptional resilience or longevity, as comparisons with general populations, and across species, can uncover conserved mechanisms of resilience [108].

Strategic research should explore microgravity and immobilisation as physiological analogues for ageing, providing insight into mechanisms of disuse-related muscle atrophy, metabolic decline, and inflammation [109]. Investigations into brown adipose tissue biology have revealed its potential role in promoting metabolic health during ageing [110]. Additionally, non-traditional interventions such as whole-body vibration therapy have shown early but still emerging promise in improving muscle mass and function among older adults with sarcopenia [111]. These approaches are presented as exploratory rather than definitive, underscoring the need for further validation. Integrating epigenetics and genotype–phenotype analyses across species and human cohorts may yield valuable translational insights. Population-level strategies for increasing physical activity include media and education campaigns, school-based interventions, environmental design, policy mandates, economic incentives, workplace programmes, and community-based settings. Taken together, these elements reflect a systems-based approach that extends beyond individual responsibility and requires coordinated public health policy [47, 112, 113].

Investment in digital health infrastructure is essential to advancing precision prevention globally. Technologies such as biosensors, wearables, non-invasive diagnostics, and artificial intelligence-driven modelling platforms must be designed to be accurate, accessible, and culturally adaptable. Recent advances underscore the transformative potential of combining human insight with machine intelligence to enhance diagnostic precision, predictive modelling, and personalised cure and care delivery [78]. Building on this foundation, there is growing recognition of the need for a multidimensional, context-sensitive framework for evaluating health, one that goes beyond traditional biomarkers to incorporate psychosocial, economic, and environmental factors, including access, affordability, motivation, cultural norms, and belief systems. The development of such a multidimensional “healthiness scale” is proposed here as a research and policy priority rather than an established metric. Developing such a comprehensive scale could support equitable, person-centred health innovation across diverse global contexts.

New institutional frameworks are needed to formally recognise emerging professional roles in health promotion, lifestyle guidance, and personalised intervention delivery. These professionals must be trained in biomedical science, behavioural coaching, digital fluency, and cultural literacy to meet the evolving demands of precision prevention. The WHO has called for competency-based education, the expansion of interdisciplinary training programmes, and strategic workforce policies to build a fit-for-purpose health workforce aligned with modern, person-centred care systems [114]. Academic programmes and certifications in precision health, digital public health, and intervention design are therefore identified as enabling mechanisms to equip professionals with the tools needed to deliver scalable, context-sensitive health promotion.

Education must also extend into communities, where social networks, cultural norms, and daily routines play a powerful role in shaping health behaviours. Community-based initiatives delivered through schools, workplaces, and digital platforms can reinforce health-promoting practices and foster sustainable behaviour change. Addressing the social determinants of health remains essential for reducing inequalities and improving population outcomes [55]. Similarly, international health system analyses emphasise the importance of embedding education and behavioural support at the community level to enhance public health and close equity gaps [113]. Policy and governance must align with the goals of precision prevention to ensure long-term impact. Intersectoral coalitions spanning health, physical activity providers, technology, finance, education, and urban planning are essential for driving systemic change. To support policy adoption, decision-makers require clear evidence on long-term social and economic benefits, including reduced healthcare expenditure, improved productivity, reduced absenteeism, and enhanced quality of life [113]. Aligning payment systems with these objectives requires a shift away from volume-based care models toward value-based approaches that incentivise prevention, early intervention, and long-term health optimisation [113].

Equity and ethics must guide innovation. Open-source platforms, ethical data governance, and public–private partnerships can promote global access and mitigate digital health divides. Intellectual property frameworks should balance innovation incentives with accessibility, particularly in low- and middle-income countries. Educational systems, especially physical education, represent foundational platforms for delivering early, context-sensitive interventions that promote lifelong physical activity and health literacy. Teachers and educators are positioned as critical local change agents.

Ultimately, precision prevention reframes healthcare as a proactive humanitarian science to foster human potential, particularly resilience and performance. Realising this vision requires a deliberate shift in scientific culture, grounded in methodological rigour, behavioural insight, cultural empathy, and public trust. With sustained investment and global collaboration, precision prevention can evolve from a promising concept into a transformative movement, one that not only advances human health and prosperity, but also addresses the growing mismatch between human biological design and rapidly changing environments.

Sport is also a powerful and currently underutilised platform for advancing the goals of precision prevention. International Federations, National Federations, and the IOC have a unique opportunity, and responsibility, to promote lifelong physical activity through their global reach and influence. Beyond elite performance, sport should be recognised and supported as a health-enhancing practice across the lifespan [48]. This is especially important for youth, who can be inspired through physical activity, fitness, and sport programmes to maintain healthy behaviours into adulthood. Initiatives that encourage continued participation after athletic careers end, particularly among women, may help counter the decline in physical activity commonly observed among former athletes.

Beyond its role in preventing non-communicable diseases and promoting healthy habits, sport, fitness, and physical activity contribute to holistic development, well-being, and quality of life across the life course. To fully harness this potential, it is essential to move beyond a narrow focus on competitive sport and adopt a “sport for all” philosophy. This inclusive approach recognises cultural diversity, social context, and accessibility, ensuring that sport initiatives are responsive to varied needs and preferences.

As a global leader in sport, the IOC plays a pivotal role in advancing physical activity and health promotion at both individual and population levels. Through strategic programmes, the IOC leverages sport to address physical inactivity and health inequity via two complementary initiatives:

1. **Athlete365:** The IOC’s platform for elite athletes, offering personalised health management tools, career transition resources, and the Athletes’ Giving Back programme, which empowers Olympians to lead community-based physical activity initiatives. In 2023, over 1,200 athlete leaders participated [50].
2. **Olympism365:** A systemic initiative embedding sport within the UN Sustainable Development Goals, aligned with the WHO Global Action Plan on Physical Activity. Launched under Olympic Agenda 2020 + 5, Olympism365 supports over 550 sport-for-development initiatives across 176 countries [50], including strategic partnerships and the allocation of USD 25 million annually through Olympic Solidarity.

Together, Athlete365 and Olympism365 reflect complementary elements of the IOC’s broader commitment to health, inclusion, and sustainable human development through sport.

At the community level, effective cross-sector collaboration requires mapping existing stakeholders and services to connect resources strategically rather than duplicate them. The fitness and physical activity sector, through its venues, professionals, and reach, already plays a substantial preventive role by enabling daily engagement in health-enhancing activity, though its full potential remains underutilised.

## Concise Recommendations and Pathways Forward

To advance precision prevention from concept to practice, coordinated action is required across research, infrastructure, workforce development, and policy. First, an international research initiative should be established to focus on systems-level health, resilience, and ageing across diverse populations, including the use of physiological analogues to better understand trajectories of decline and adaptation. Parallel investment is needed in equitable digital health infrastructure, including biosensors, wearables, and artificial intelligence models that are interoperable, culturally sensitive, and scalable across resource settings. To support a more holistic evaluation of health, the development of a multidimensional “healthiness scale” is recommended, integrating psychosocial, environmental, economic, and cultural indicators alongside biological measures.

Effective implementation will also require workforce transformation. New professional roles and training pathways in precision health should be developed, integrating biomedical, behavioural, digital, and cultural competencies, and extending beyond traditional clinician-centred models of care. Community-based health education should be expanded through schools, workplaces, sport organisations, and digital platforms to reinforce lifelong engagement in physical activity and healthy behaviours. At the system level, policy and payment mechanisms should be aligned with prevention and long-term outcomes, including the adoption of value-based care models that incentivise sustained health rather than episodic disease treatment.

Finally, intersectoral collaboration must be strengthened across health, sport, education, urban planning, and finance to create supportive environments for active living. Sport and fitness systems should be leveraged as strategic platforms for lifelong physical activity participation, while prevention should be embedded early within education and public health infrastructure. Across all actions, ethical and equitable innovation must be prioritised, including commitments to open science, data stewardship, and meaningful access for low- and middle-income countries.

## Global Alliance for the Promotion of Physical Activity—A Paradigm Shift

The GA@PA represents a coordinated, implementation-oriented initiative to embed the principles of precision prevention into global health policy and practice. In response to alarming trends, such as approximately 28% of adults and 81% of adolescents worldwide having physical activity levels below recommended thresholds [115], and emerging data showing that over 2.6 billion people are now affected by overweight or obesity [1], the Alliance underscores the urgent need to shift toward proactive, preventive strategies. It addresses physical inactivity as a critical determinant of chronic disease, reduced resilience, and escalating healthcare costs. By supporting the implementation of expert strategies such as the WHO Global Action Plan on Physical Activity and forging partnerships that prioritise evidence-based, locally adapted, and scalable interventions [47], the Alliance seeks to operationalise prevention on a global scale and provides a framework for implementation strategies (Fig. 2). Together, these efforts reflect a proposed paradigm shift from treating disease to cultivating health through data-informed, equitable, and systems-oriented approaches.

Bringing together over 130 institutions across medicine, sport, fitness, physical activity, public health, and governance, the Alliance promotes a unified, multisectoral conceptual framework and related strategy aligned with the core tenets of precision prevention [1]. Its mission is to shift health systems from reactive treatment to proactive health enhancement by embedding physical activity, sports and exercise science and medicine, and personalised interventions into daily life and healthcare frameworks. Central to the GA@PA’s approach is the promotion of policies that prioritise prevention, support healthcare providers and allied health professionals in prescribing physical activity and exercise, and adapt physical activity and exercise strategies to individual, cultural, and environmental contexts. This reflects precision prevention’s emphasis on personalised, context-sensitive interventions addressing both biological and social health determinants.

The Alliance also leverages technology by collaborating with technology developers, healthcare providers, and educators to integrate wearables, gamification, and digital tools into health promotion strategies. Within this framework, continuous monitoring, real-time data, and AI-supported analytics are positioned as enabling tools to inform personalised care rather than as standalone solutions. Moreover, the GA@PA recognises that sustained behaviour change requires cultural relevance and community engagement. By utilising athletes and other role models as advocates and developing structured advocacy platforms, it aims to foster broader societal shifts in how physical activity and prevention are valued, consistent with behavioural science principles. With a defined governance structure, performance indicators, and a roadmap involving global summits and stakeholder engagement, the GA@PA offers a scalable implementation framework for advancing precision prevention worldwide. Its vision extends beyond reducing physical inactivity to embedding health-enhancing behaviours into the fabric of society, with explicit attention to equity, accessibility, and long-term sustainability.

In essence, the GA@PA seeks with this conceptual paper to operationalise the principles of precision prevention by bridging science, technology, culture, and policy to support adaptive capacity, disease prevention, and population health. At the Sports, Medicine and Health Summit (26–28 June 2025, Hamburg, Germany), members and partners of the GA@PA renewed their commitment by endorsing the 2025 Hamburg Declaration on Precision Prevention and Personalised Health Enhancement (see Supplementary Information).

## Approach and Limitations

This article is presented as a narrative, practice-informed conceptual framework rather than a systematic or scoping review. The GA@PA framework was developed through an iterative process involving expert consultation, interdisciplinary dialogue, and synthesis of existing evidence across physical activity, exercise science and medicine, behavioural science, digital health, and public health policy. Evidence cited throughout the article is drawn from peer-reviewed literature and selected to illustrate key concepts relevant to precision prevention and physical activity promotion, rather than to provide an exhaustive or quantitatively weighted appraisal of all available studies.

As such, the framework reflects expert consensus and integrative interpretation rather than formal evidence grading, meta-analysis, or Delphi-based agreement. This approach was intentionally chosen to support translation across diverse global contexts, where rigid, single-discipline models may lack feasibility or relevance.

Several limitations should be acknowledged. First, the breadth of the framework necessitates variable depth across domains, and some areas of precision prevention remain supported by emerging rather than definitive evidence. Second, implementation pathways and cost-effectiveness will vary substantially across health systems, cultures, and resource settings and require local adaptation and empirical evaluation. Third, although the framework emphasises global equity, the authorship and cited evidence predominantly reflect high- and middle-income settings, underscoring the need for expanded research capacity and leadership in under-represented regions.

Accordingly, the framework should be viewed as a testable and adaptable foundation for future research, implementation studies, and policy development, rather than as a prescriptive or finalised model of precision prevention.

## Conclusions

Precision prevention marks a proposed shift in healthcare, reframing it as a proactive, adaptive approach focused on optimising resilience, personalising care, and preventing disease across the lifespan. The core challenge remains clear: global trends of declining physical activity, increasing unhealthy body composition (including high body fat and low muscle mass), and widening health inequities continue to drive the burden of non-communicable diseases. Without altering this trajectory, pressures on individuals, health systems, and economies are expected to intensify. This paper has outlined a narrative, implementation-oriented conceptual framework integrating omics, artificial intelligence, behavioural science, education, and systems medicine into a unified, context-aware approach to address this challenge. However, technological and biological advances alone are insufficient unless they enable sustained behaviour change. Achieving this requires systemic transformation across sectors, including education, physical activity provision, infrastructure, policy, healthcare delivery, digital innovation, and economic incentives. A durable paradigm shift depends on environments and systems that make healthy behaviours accessible, supported, and rewarding for diverse populations.

At its core, precision prevention moves beyond generic recommendations to support targeted interventions shaped by dynamic health data, cultural relevance, and individual needs. Tools such as digital twins, biosensors, and predictive analytics may support real-time health optimisation, while personalised strategies for physical activity and exercise, nutrition, sleep, and mental well-being provide potentially scalable approaches grounded in existing evidence.

However, the promise of precision prevention can only be realised through systemic transformation. This involves more than incremental improvement; it requires a re-examination of how health is defined, delivered, and prioritised across society. Such transformation includes shifting from reactive, disease-centred models toward proactive approaches emphasising health promotion and early intervention. It also requires embedding personalised prevention strategies into everyday settings, such as early childhood education, schools, workplaces, urban environments, and public policy, so that prevention extends beyond clinical contexts. Equity remains foundational, requiring that innovations are accessible, culturally responsive, and ethically governed, particularly in underserved or resource-limited communities.

The GA@PA exemplifies one pathway toward this transformation by mobilising cross-sector collaboration to embed health-enhancing behaviours into policy and daily life. As the Alliance has renewed its commitment through the 2025 Hamburg Declaration [116], the global health community stands at a critical juncture. With sustained investment, scientific rigour, cultural sensitivity, and coordinated implementation, precision prevention can be evaluated, refined, and potentially scaled as a global movement to enhance human potential, reduce disease burden, and support resilient health systems.

## Supplementary Information

Below is the link to the electronic supplementary material.

Supplementary file1 (DOCX 50 KB)

## Declarations

### Availability of Data and Material

Data sharing is not applicable to this article as no datasets were generated or analysed during the current study.

### Evidence Base and Scope

The evidence cited in this article reflects peer-reviewed literature selected to support key concepts relevant to precision prevention and the promotion of physical activity. While the article does not aim to provide an exhaustive or systematically weighted review of all available studies, references were chosen to reflect established findings, representative examples, and emerging evidence across multiple disciplines. The framework is intended to stimulate further context-sensitive empirical testing rather than to assert definitive causal claims.

### Author Affiliation Statement

Given the international and multidisciplinary nature of this consortium, co-authorship should not be interpreted as indicating direct collaboration between all authors and all represented institutions. Author affiliations are provided solely to identify the institutional appointments of individual contributors.
