# Supplementary material for: Precision Prevention for Health: The Global Alliance Framework for the Promotion of Physical Activity
Source: Sports Med. 2026 Sep 21;56(12):2929–54. doi: 10.1007/s40279-026-02495-4 (PMC13759645; doi:10.1007/s40279-026-02495-4)
Supplement: Supplementary file 1 [file 40279_2026_2495_MOESM1_ESM.docx]

**Precision Prevention for Health: The Global Alliance Framework for the Promotion of Physical Activity**

**Supplementary Information:**

**2025 Hamburg Declaration on Precision Prevention for Health**

**A Global Alliance Framework for the Promotion of Physical Activity**

**Adopted in Hamburg, Germany, 27 June 2025**

In recognition of the urgent need to transform global health systems, from reactive disease management to proactive, personalised health promotion, and in response to the rising burdens of chronic disease, physical inactivity, health inequities, and climate-related health threats, we, the members of the Global Alliance for the Promotion of Physical Activity, commit to the following principles:

1. Establish Precision Prevention as a Global Health Imperative

We commit to:

1. Integrate precision prevention into national and global health strategies by harnessing multi-omics technologies (e.g., genomics, epigenetics, proteomics), AI-powered analytics, and wearable biosensors to enable tailored, predictive interventions.
2. Elevate physical activity as a central pillar of health, adopting the 10-Pillar Model to address Exercise Deficiency Syndrome (EDS) through personalised, context-aware approaches.
3. Apply evolutionary medicine principles to correct the mismatch between modern environments and human biology, promoting movement, nutrition, and resilience as essential to healthy ageing and longevity.

**2. Promote Equity in Precision Health Innovation**

We pledge to:

1. Bridge the digital and research divide by expanding access to affordable sensors, mobile health tools, and culturally relevant interventions, especially in underrepresented regions such as Africa and Latin America.
2. Invest in localised, participatory research to address the dual burden of undernutrition and obesity, co-developing solutions with communities historically marginalised in global health agendas.
3. Uphold ethical governance of AI and data, ensuring privacy, transparency, and equitable access to emerging precision health technologies.

**3. Mobilise Cross-Sector Action for Health-Enabling Environments**

We will:

1. Foster collaboration across healthcare, education, sport, urban design, and transport to create environments that make physical activity the easy, accessible choice.
2. Reorient policy and economic incentives to reward prevention, including value-based care models and tax or fiscal levers supporting healthy schools, workplaces, and cities.
3. Engage the sport and fitness sectors as drivers of change, scaling programmes such as the IOC’s Olympism365 and Athlete365 to bridge elite athlete insights with community-based health promotion.

**4. Enable Lifelong Health Through Education and Digital Innovation**

We commit to:

1. Transform health professional training, embedding physical activity counselling, nutrition literacy, and behavioural coaching into standard medical curricula, positioning physical activity as a vital sign.
2. Implement digital twins and continuous monitoring to optimise individualised strategies for sleep, mental health, and environmental resilience (e.g., heat adaptation and climate stress protocols).
3. Engage young people and communities through co-creation and participatory design, using sport, social platforms, and gamified tools to sustain positive health behaviours.

**5. Measure Impact and Accelerate Scaling**

We will:

1. Develop a multidimensional “healthiness scale” that integrates biological, psychological, social, and environmental indicators to track resilience, equity, and overall well-being.
2. Launch a global research consortium to study high-performing outliers (e.g., centenarians, elite athletes), translating insights into scalable precision prevention solutions.
3. Convene biennial summits to evaluate progress, exchange evidence-based strategies, and align efforts with the UN Sustainable Development Goals (SDGs).

**Conclusion**

By endorsing this Declaration, we reaffirm our shared commitment to redefine healthcare as a proactive, personalised, and prevention-focused system. We call on governments, industries, academia, and civil society to join this global movement, ensuring that precision prevention becomes the foundation of 21st-century health, equity, and resilience.

**Signed in Hamburg, Germany, 27 June 2025, by the Members of the Global Alliance for the Promotion of Physical Activity.**
